# Household catastrophic health expenditure for COVID-19 during March-August 2021, in South India: a cross-sectional study

**DOI:** 10.1186/s12889-022-14928-6

**Published:** 2023-01-06

**Authors:** Elumalai Rajalakshmi, Akhil Sasidharan, Bhavani Shankara Bagepally, Muthusamy Santhosh Kumar, Ponnaiah Manickam, T. S. Selva Vinayagam, P. Sampath, K Parthipan

**Affiliations:** 1 ICMR-National Institute of Epidemiology, Chennai, India; 2 Health Technology Assessment Resource Centre, ICMR-National Institute of Epidemiology, Tamil Nadu Housing Board, Phase I and II, Ayapakkam, Chennai, India; 3 Tamil Nadu Directorate of Public Health and Preventive Medicine, Chennai, India

**Keywords:** Household catastrophic health expenditure, COVID-19, Out of pocket expenditure

## Abstract

**Background:**

The Coronavirus disease 2019 (COVID-19) pandemic increased the utilisation of healthcare services. Such utilization could lead to higher out-of-pocket expenditure (OOPE) and catastrophic health expenditures (CHE). We estimated OOPE and the proportion of households that experienced CHE by conducting a cross-sectional survey of 1200 randomly selected confirmed COVID-19 cases.

**Methods:**

A cross-sectional survey was conducted by telephonic interviews of 1200 randomly selected COVID-19 patients who tested positive between 1 March and 31 August 2021. We collected household-level information on demographics, income, expenditure, insurance coverage, direct medical and non-medical costs incurred toward COVID-19 management. We estimated the proportion of CHE with a 95% confidence interval. We examined the association of household characteristics; COVID-19 cases, severity, and hospitalisation status with CHE. A multivariable logistic regression analysis was conducted to ascertain the effects of variables of interest on the likelihood that households face CHE due to COVID-19.

**Results:**

The mean (95%CI) OOPE per household was INR 122,221 (92,744–1,51,698) [US$1,643 (1,247–2,040)]. Among households, 61.7% faced OOPE, and 25.8% experienced CHE due to COVID-19. The odds of facing CHE were high among the households; with a family member over 65 years [OR = 2.89 (2.03–4.12)], with a comorbid individual [OR = 3.38 (2.41–4.75)], in the lowest income quintile [OR = 1.82 (1.12–2.95)], any member visited private hospital [OR = 11.85 (7.68–18.27)]. The odds of having CHE in a household who have received insurance claims [OR = 5.8 (2.81- 11.97)] were high. Households with one and more than one severe COVID-19 increased the risk of CHE by more than two-times and three-times respectively [AOR = 2.67 (1.27–5.58); AOR = 3.18 (1.49–6.81)].

**Conclusion:**

COVID-19 severity increases household OOPE and CHE. Strengthening the public healthcare and health insurance with higher health financing is indispensable for financial risk protection of households with severe COVID-19 from CHE.

**Supplementary Information:**

The online version contains supplementary material available at 10.1186/s12889-022-14928-6.

## Introduction

The Coronavirus disease 2019 (COVID-19) pandemic affected many countries globally with a multi-dimensional impact on every aspect of life, including the economy, social life, politics, technology, environment and health care [1, 2]. During the COVID-19 pandemic, countries have implemented various measures to mitigate the virus transmission and prevent overwhelming health systems. The Government of India imposed a nationwide lockdown to prevent the spread during the first wave, and it enforced state-level restrictions during the second wave [3, 4]. These restrictions negatively impacted people's livelihood, particularly those from marginalized communities [5]. The rapid emergence of COVID-19 cases overwhelmed the Indian healthcare system, and the government had to act under significant uncertainty and severe economic and social pressure [4, 6]. Though most COVID-19 patients are asymptomatic or experienced mild symptoms [7], the elderly and people with comorbidities are at higher risk of hospitalization and death [8]. Patients with severe diseases are more likely to be hospitalized in intensive care units (ICU) or require more extended hospital stays, leading to higher out-of-pocket expenditure (OOPE) and catastrophic health expenditures (CHE) [9]. To reduce the OOPE, the government capped the price of testing for SARS-CoV-2, ambulance services, and bed charges for ICU and non-ICU beds. However, there were no mechanisms to guarantee that private hospitals followed the notification guidelines [10].

The Government spending on health in India is only about 1.5% of GDP [11]. India's health sector is characterized by low government expenditure on health, high OOPE and low financial protection from adverse health events [12]. The majority of the Indian population seeks care from the private sector, with two-thirds of the total health expenditure being out-of-pocket [13]. Indian health care system, already distressed in both capacity and quality of service due to persistently low public spending on health, was further weakened by the pandemic. The increased demand-side utilization led to overcrowding and overwhelmed the healthcare system [14, 15]. The overburdening of public hospitals often diverts healthcare seekers to get treatment from the private sector, which is usually costlier [16]. The 75^th^ round of the national sample survey (NSSO) on social consumption of health, 2017–18, showed that nearly 60% of all hospitalizations and the private sector deliver about 70% of outpatient services in India [17]. The Ayushman Bharat—Pradhan Mantri Jan Arogya Yojana (AB-PMJAY), a national health insurance scheme implemented in 32 states and union territories, rolled out COVID-19 packages targeting poor and vulnerable families [18]. Several other union and state governments sponsored health insurance schemes purchased care from the private sector [19]. In Tamil Nadu, through the Chief Minister's Comprehensive Health Insurance Scheme (CMCHIS), the government included COVID-19 packages without any co-payments for obtaining care from the empanelled hospitals [20]. It is well-documented from prior studies that households may face financial catastrophe and deprivation due to increased OOPE [21–24]. Literature shows how the COVID-19 pandemic made families vulnerable to CHE due to health systems' growing dependence on OOPE [25, 26]. Hence, there is a need to generate information on OOPE and CHE due to COVID-19 in India. In this context, we conducted this study to estimate the household OOPE for COVID-19 treatment and the proportion of households that experienced CHE in Tamil Nadu. Further, we examined the association of household characteristics with CHE and whether COVID-19 severity was associated with an increase in catastrophic expenditure for the households, also calculated concentration curves and indices for equity analyses.

## Methods

We conducted a cross-sectional study in Tamil Nadu during November and December 2021. We obtained a list of RT-PCR confirmed COVID-19 cases between 1 March and 31 August 2021 from the Directorate of Public Health and Preventive Medicine, Tamil Nadu. After sorting the list chronologically according to the date reported as COVID-19 positive, we selected COVID-19 patients using simple random sampling using computer-generated random numbers. We estimated a sample size of 600, based on the assumptions of 15% of households incurring CHE (study conducted by ICMR-NIE in Vellore district, Tamil Nadu), 5% absolute precision, 95% confidence level, design effect of 2, and 40% non-response. Subsequently, we inflated the sample size to 1200 to allow a refusal rate of 50% in the telephonic survey. They were randomly selected from the line list of 17,58,686 COVID-19 positive cases with contact details. We collected the household income and expenditure related information and patient information for all family members infected with COVID-19 in the same period. Hence, the unit of analysis is the 'household' in the current study. We further excluded 40 households from the 787 interviewed because they either did not report their annual income and expenses or were unaware of their COVID-19 hospitalization costs.

We contacted the selected individuals using mobile phone numbers provided in the line list. Interviews were scheduled as per the convenience of the respondent. If the chosen individual did not pick up the call on the first attempt, three more attempts were made the same day before declaring the individual a non-respondent. We excluded patients whose phones were turned off, unavailable, or who became unavailable after three attempts at calling. We also excluded patients who had incorrect contact information, refused to participate, were unable to communicate in English or Tamil, or had died and their family members refused to participate. Interviewers explained the study to the respondents, who included all study household members and obtained their informed oral consent. We collected data using a pretested structured questionnaire available in English and Tamil. We collected data through Open Data Kit (ODK) Collect, an open-source Android application [27].

We collected information about the patient such as age, gender, residential address, and other details such as level of education, type of employment, household size, number of earning members in the household, including pensioners, average monthly income and expenditure of the household, and information on the head of the household, including education and occupation. We collected data on clinical symptoms, pneumonia, and history of comorbidity during the active phase of the illness to categorize the severity and the outcome at the time of the interview. We also collected information on COVID-19 conditions for estimating direct medical and non-medical costs, type of containment mechanism—home or institutional quarantine, private/government hospitalization status, duration of hospital stay, registration fees, consultation fees, lab test charges, medicine costs, and other direct medical expenses if any. Direct non-medical expenses such as food, accommodation, and transportation were collected for the patient and the caregiver. We collected data on household OOPE for COVID-19 from the date of RT-PCR confirmation until recovery, which includes information on financial coping strategies such as type of insurance scheme, mortgage of jewels, selling of any vehicle or property for COVID-19 treatment. The quality of the data collection was maintained by the intensive training of interviewers with the study questionnaire. We used an interviewer guide to ensure standardized techniques for high-quality data collection. The data collection process was monitored using a day-wise status log of interviews maintained on a Google sheet, and we conducted review meetings every alternate day.

Data were analyzed using Stata version 16 [28]. We classified the districts by Human Development Index (HDI) zones into very high, high, medium, and low HDI. COVID-19 Severe cases were defined as patients on oxygen support and under ICU admission. Distress health financing is a situation when a household has to borrow money or sell its property or assets or when it gets contributions from friends or relatives to meet its health care expenses [29]. Household characteristics were described using frequencies and percentages. Direct medical and direct non-medical costs were expressed as mean (SD) or median (IQR). OOPE is expressed as the median [inter quartile range (IQR)]. All costs are reported in Indian rupee (₹) and US dollar ($) (Conversion factor, 1 US$ = 74.37₹). We adopted the World Health Organisation (WHO) definition of health expenditure greater than or equal to forty per cent of a household's non-subsistence annual income for CHE [30]. We used 10%, 25% thresholds for subsistence income and 10% of total annual income for sensitivity analysis in calculating CHE [31]. We estimated the proportion of CHE (with 95% CI). Pearson’s chi-squared test for association was used to identify whether there is statistical significance between the categorical variables. The variables that showed significant associations in the univariate analysis were used in the bivariate analysis to understand the risk of CHE for the associated variables. We performed a multivariable logistic regression analysis to determine the effects of number of associated variables with households facing CHE. In addition, we used concentration curves and indices to perform equity analysis.

As per the National ethical guidelines for ethics committees reviewing biomedical and health research during COVID-19 pandemic by the Indian Council of Medical Research in April 2020, If written consent is not possible (COVID-19 patients), consent could be given orally/ use electronic methods to document and record. The study protocol was reviewed and approved by the institutional human ethics committee of ICMR-National Institute of epidemiology (NIE/IHEC/202107–04), Chennai for telephonic survey targeting adults affected by COVID-19.. Oral/verbal consent was obtained and recorded before the start of each telephonic interview. We got permission from the State public health department of Tamil Nadu to conduct this study.

## Results

**Fig. 1:**
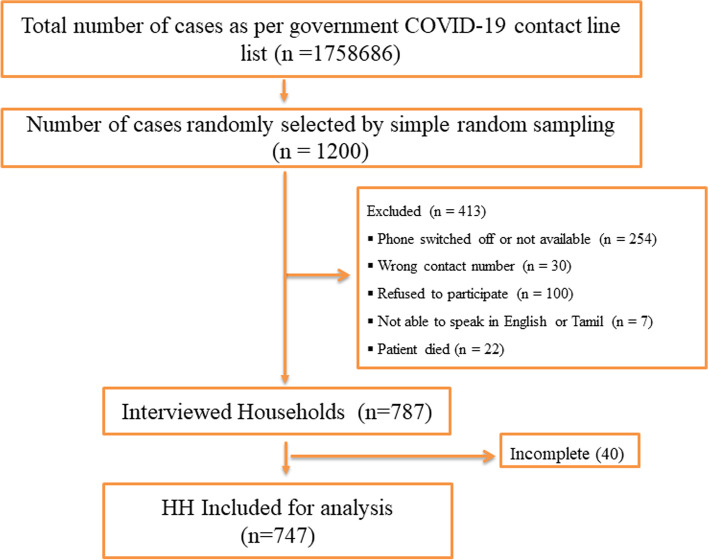
Flowchart of Study Selection

The study participant/household selection is shown in Fig. 1. Out of 1200 randomly selected, we excluded 437 patients: those who had their phones switched off or not available, 30 with wrong contact numbers, 100 who refused to participate, seven who could not speak in English or Tamil and 22 who had died and their family members declined to participate. Seven hundred and forty-seven households were included for the final analysis.

### Household characteristics of COVID-19 patients

The general characteristics of households analysed in this study are shown in Table 1. The number of people per household ranged from 1 to 16, with a median (IQR) of 4 (2). The number of COVID-19 patients in a household ranged from one to seven, with a median (IQR) of one (1). Nearly half (42.3%) of the participating households were from districts with very high HDIs, and only 2.5% were from districts with low HDIs. More than half (51.1%) reported having unstable employment. One-third of the households (66.5%) had only one earning member, and more than a quarter (31.3%) of the households were living on rent. A quarter of the households (25.3%) had a family member over 65 years of age, and one-third of the households (34.3%) had a family member with any comorbidity. Less than half of the households (43.2%) had only one case of COVID-19. Nearly half of the households included had visited health facilities more than once and had had an episode of hospitalization. About one-third (30%) of households have used a private hospital at least once. One in every two households was facing OOPE (62%) and distress financing (52%). A severe COVID-19 patient was found in three out of every twenty households. One in eight households self-reported of having some form of insurance towards health; among those, 40% of the households received any co-payments or reimbursements.

**Table 1:** Catastrophic Health Expenditure and its association with household characteristics

Characteristics	*N* = 747 (%)	With CHE^a^ (*n* = 193)	*p*-value ^b^
Place of residence			
Urban	306 (41)	80 (41.5)	0.873
Rural	441 (59)	113 (58.5)	
Family type			
Joint	220 (29.5)	63 (32.6)	0.259
Nuclear	527 (70.5)	130 (67.4)	
Household’s district HDI zone			
Very High	319 (42.7)	78 (40.4)	0.774
High	118 (15.8)	30 (15.5)	
Medium	290 (38.8)	81 (42.0)	
Low	20 (2.7)	4 (2.1)	
Household size			
1 -2	133 (17.8)	36 (18.7)	0.752
3- 4	416 (55.7)	103 (53.4)	
> 4	198 (26.5)	54 (28)	
Household head education			
Below elementary level	80 (10.7)	16 (8.3)	0.272
Primary education	54 (7.2)	19 (9.8)	
Secondary & higher secondary	224 (30)	57 (29.5)	
College education	389 (52.1)	101 (52.3)	
Household head employment status			
Unemployed	54 (7.2)	15 (7.8)	0.933
Unstable employment	378 (50.6)	98 (50.8)	
Stable employment	315 (42.2)	80 (41.5)	
Household Earning members			
None	8 (1.1)	2 (1.0)	0.010*
One	497 (66.5)	147 (76.2)	
Two	207 (27.7)	39 (20.2)	
> 2	35 (4.7)	5 (2.6)	
Household Income quintile			
First	143 (19.1)	52 (26.9)	0.021*
Second	126 (16.9)	25 (13.0)	
Third	171 (22.9)	44 (22.8)	
Fourth	127 (17)	29 (15)	
Fifth	180 (24.1)	43 (22.3)	
Household living in rented houses			
Yes	234 (31.3)	58 (30.1)	0.658
No	513 (68.7)	135 (69.9)	
Households reported as having health Insurance			
Yes	95 (12.7)	43 (22.3)	< 0.001 ^c^*
No	648 (86.7)	150 (77.7)	
Do not know	4 (0.6)	0	
Households receiving Insurance payment			
Yes	34 (4.6)	22 (11.5)	< 0.001*
No	708 (95.4)	170 (88.5)	
Households having a family member over 65 years of age			
Yes	189 (25.3)	80 (41.5)	< 0.001*
No	558 (74.7)	113 (58.5)	
Households with at least one comorbid individual			
Yes	256 (34.3)	107 (55.4)	< 0.001*
No	491 (65.7)	86 (44.6)	
Number of Covid-19 Patients per Household			
1 -2	600 (80.3)	141 (73.1)	0.009*
3- 4	134 (17.9)	46 (23.8)	
> 4	13 (1.7)	6 (3.1)	
Households with more than one Covid-19 infection			
Yes	323 (43.2)	108 (56)	< 0.001*
No	424 (56.8)	85 (44)	
Number of severe Covid-19 cases per household			
None	624 (83.5)	111 (57.5)	< 0.001*
One	56 (7.5)	33 (17.1)	
Two or more	67 (9)	49 (25.4)	
Households with more than one Healthcare facility visit			
Yes	379 (50.7)	130 (67.4)	< 0.001*
No	368 (49.3)	63 (32.6)	
Households with at least one episode of hospitalization for COVID-19			
Yes	343 (45.9)	164 (85)	< 0.001*
No	404 (54.1)	29 (15)	
Households with at least one private hospital visit			
Yes	226 (30.3)	142 (73.6)	< 0.001*
No	521 (69.7)	51 (26.4)	
Households with distress financing			
Yes	388 (51.9)	120 (62.2)	< 0.001*
No	359 (48.1)	73 (37.8)	
Household facing OOPE			
Yes	461 (61.7)	175 (90.7)	< 0.001*
No	286 (38.3)	18 (9.3)	

*Abbreviation*: *CHE* Catastrophic health expenditure, *OOPE* Out of pocket expenditure

^a^Households who met the definition of CHE

^b^χ^2^Test comparing Households which was not found to have CHE with households reporting CHE

^c^Fisher’s Exact Test

^*^p-value is significant

### COVID-19 patient characteristics

The mean age of the participants was 41.04 ± 17.69 years, and 52% were males. 4% of the males reported smoking tobacco, and 8% reported using alcohol. 16.4% of the patients were asymptomatic. Fever (70%) was the most prevalent symptom, followed by cough (41%), myalgia (34%), sore throat (29%), and runny nose (28%). More than one-third of the patients reported a loss of smell (37%) and loss of taste (39%). Only 13% of patients reported shortness of breath. Diabetes (17.5%) and hypertension (13.2%) were the most commonly reported comorbidities. One-fifth of the patients had been hospitalised at least once in private hospitals (19.5%) and government hospitals (17.35%). The average number of days spent in a private hospital was higher, with a mean hospital duration of 7.87 ± 4.72 days, median (IQR) 7 (5) compared to a government hospital with a mean hospital duration of 6.88 ± 4.14, median (IQR) of 7 (4). The mean ICU duration was 6.27 ± 5.40, with a median (IQR) of 5 (4) days.

### Out of pocket expenditure (OOPE)

The mean (95% CI) OOPE per household was INR 122,221 (92,744–151,698) [US $1,643 (1,247–2,040)], and the median (IQR) values were INR 3,500 (41,300); [US $47 (555)]. The mean (95% CI) household’s annual income and total health expenditure were INR 397,566 (362,167–432,964), INR 116,622 (87,558–145,686) [US $5,346 (4,870–5,822), $1568 (1,177–1,959)], and the median (IQR) values were INR 300,000 (276,000), INR4,000 (4,13,000); [US $ 4,034 (3,711), $54 (5,533]. The mean (95% CI) direct health expenditure and direct non-medical expenditure were respectively INR 115,356 (86,143–144,569), INR 8,292 (6,352–10,231); [US $ 1,551 (1,158–1,944), 111 (85–138)] and the median (IQR) values were INR 1,350 (41,000), 0 (0); [US $18 (551), 0]. (Supp Table 2).

### Catastrophic health expenditure (CHE) and its determinants

Households with CHE due to COVID-19 illness was 25.84% (95% CI: 22.69–29.0), with 36.36% (95% CI: 28.38–44.34%) in the lowest income quintile and 19.84% (95% CI: 12.78–26.90) in the highest income quintile. Households in the rural areas, coming from the lowest income quintile, having only one earning member, had a higher proportion of CHE. Households with the head of households having an unstable employment status or education below the elementary level had a higher proportion of CHE. Households having at least one person over 65 years of age, one person with comorbidity or one person with more than one COVID-19 infection had higher proportions of CHE. Households with at least one episode of hospitalization and at least one private hospital visit experienced higher CHE proportions. Around 2.5 percent of the households reported a negative non-subsistence income (Table 1).

The main drivers for CHE was severe COVID-19 infection, with one severe COVID-19 case per households having an OR of 6.63 [(3.75–11.73), *p*-value < 0.001], and two or more severe COVID-19 cases having an OR of 12.58 [(7.06–22.42, *p*-value < 0.001]. Other predictor variables included admission to a private hospital [OR = 15.58 (10.49–23.13), *p*-value < 0.001], at least one episode of hospitalisation [OR = 11.85 (7.68–18.27), *p*-value < 0.001], facing OOPE [OR = 11.0 (5.46–15.22), *p*-value < 0.001], having a family member over 65 years of age [OR = 2.89 (2.03–4.12), *p*-value < 0.001] and households with at least one comorbid individual [OR = 3.38 (2.41–4.75), *p*-value < 0.001]. Households that self-reported having a health insurance [OR = 2.75 (1.76 – 4.28), *p*-value < 0.001] and households with insurance who have received co-payments/reimbursements also had an increased odd of facing CHE [OR = 5.8 (2.81–11.97), *p*-value < 0.001] (Table 2).

**Table 2:** Association between facing CHEs and household characteristics

Characteristics	Crude OR (95% CI)	*p*-value (Crude OR)	Adjusted OR (95% CI)	*p*-value (Adjusted OR)
Household Earning members				
None	reference group			
One	1.26 (0.25 – .31)	0.779	1.69 (0.21 – 13.74)	0.626
Two	0.7 (0.14 –3.58)	0.667	0.88 (0.10 – 7.59)	0.908
> 2	0.47 (0.08 – 0.21)	0.465	0.54 (0.05 – 6.28)	0.62
Household Income quintile				
First	1.82 (1.12 – 2.95)	0.015*	12.43 (5.25 – 9.47)	< 0.001*
Second	0.79 (0.45 – 1.38)	0.402	3.80 (1.59 – 9.18)	0.003*
Third	1.1 (0.68 – 1.79)	0.690	2.87 (1.34 – 6.15)	0.007*
Fourth	0.94 (0.55 – 1.61)	0.830	2.47 (1.12 – 5.46)	0.026*
Fifth	reference group			
Households reported as having health Insurance				
Yes	2.75 (1.76 – 4.28)	< 0.001*	2.91 (1.23 – 6.87)	0.015*
No	reference group			
Households receiving Insurance payment				
Yes	5.8 (2.81 – 11.97)	< 0.001*	0.51 (0.16 – 1.58)	0.241
No	reference group			
Household having a family member over 65 years of age				
Yes	2.89 (2.03 – 4.12)	< 0.001*	1.62 (0.95 – 2.74)	0.075
No	reference group			
Households with at least one comorbid individual				
Yes	3.38 (2.41 – 4.75)	< 0.001*	1.59 (0.95 – 2.68)	0.077
No	reference group			
Number of COVID-19 Patients per Household				
1 -2	reference group			
3- 4	1.7 (1.14 – 2.55)	0.010*	1.61 (0.80 – 3.23)	0.180
> 4	2.79 (0.92 – 8.44)	0.069	1.68 (0.35 – 8.01)	0.514
Households with more than one COVID-19 infection				
Yes	2.00 (1.44 – 2.79)	< 0.001*	1.87 (0.93 – 3.74)	0.079
No	reference group			
Number of severe COVID-19 cases per household				
None	reference group			
One	6.63 (3.75 – 11.73)	< 0.001*	2.67 (1.27 – 5.58)	0.009*
Two or more	12.58 (7.06 – 22.42)	< 0.001*	3.18 (1.49- 6.81)	0.003*
Households with more than one Healthcare facility visit				
Yes	2.53 (1.80 – 3.57)	< 0.001*	0.4 (0.19 – 0.81)	0.01*
No	reference group			
Households with at least one episode of hospitalisation				
Yes	11.85 (7.68 – 18.27)	< 0.001*	3.87 (2.17 – 6.92)	< 0.001*
No	reference group			
Households with at least one private hospital visit				
Yes	15.58 (10.49–23.13)	< 0.001*	8.41 (4.76 – 14.84)	< 0.001*
No	reference group			
Household facing OOPE				
Yes	9.11 (5.46 – 15.22)	< 0.001*	2.51 (1.28 – 4.89)	0.007*
No	reference group			

Variable (s) entered in logistic regression: Household Earning members Household_Income_Quintiles, Households reported as having health Insurance, Households receiving Insurance payment, Household having a family member over 65 years of age, Households with at least one comorbid individual, Number of COVID-19 Patients per Household, Households with more than one COVID-19 infection, Number of severe COVID-19 cases per household, Households with more than one Healthcare facility visit, Households with at least one episode of hospitalisation, Households with at least one private hospital visit, Household facing OOPE

Hosmer and Lemeshow test for goodness of fit, χ2 = 6.978 (p = 0.539). Classification = 87.1%

Nagelkerke R^2^ = 0.562, Pseudo R^2^ = 0.423, McFadden's R^2^ = 0.423, McKelvey and Zavoina's R^2^ = 0.588, Efron's R^2^ = 0.481

*Ref* Referent, *CI* Confidence intervals

^*^p-value is significant

A multivariable logistic regression analysis was performed to ascertain the effects of household earning members, household income status, health insurance status, households receiving insurance payment, number of COVID-19 patients per household, household having a family member over 65 years of age, household with at least one comorbid individual, household with more than one COVID-19 patient, number of severe COVID-19 cases in a household, household with at least one hospitalisation for COVID-19 and household with at least one private hospital for COVID-19 visit on the likelihood that households have CHE. The logistic regression model was statistically significant, with Hosmer–Lemeshow goodness of fit, p > 0.05. The model explained 56.2% (Nagelkerke R^2^) of the variance in facing CHE and correctly classified 87.1% of cases. Having one severe COVID-19 patient in a household increases the risk of CHE by more than two times [AOR = 2.67 (1.27 – 5.58)] and having more than one COVID-19 patient in a household increases the risk by more than three times [AOR = 3.18 (1.49 – 6.81)] (Table 2).

### Sensitivity analysis for CHE thresholds

Using 10% and 25% thresholds for subsistence income for calculating CHE, 36.81% (33.34–40.28) and 29% (95%CI: 25.79–32.31) of the households faced CHE. We also found that 196 households (26.24%, 95%CI: 23.3–29.40) had faced CHE due to COVID-19 while considering CHE as total health expenses of more than 10% of total annual income.

### Concentration curves and indices

The concentration curve and index value of 0.384 reveal inequality in household income. There was more inequality in household subsistence income when household income was taken into account. This was shown by an index value of 0.456. There was minimal inequality for severe COVID-19 disease in households and for annual or subsistence income. There was relative equality in OOPE based on household income. An index value of 0.576 showed a severe inequality in OOPE in relation to the number of severe COVID-19 patients in the household. 80% of the OOPE was made up of households with a high proportion of severe COVID-19 cases (Supp Fig. 1, Supp Table 1).

## Discussion

Our study findings reveal that more than half of the households faced OOPE, one-fourth of the total households experienced CHE, and half of the households had distress financing due to COVID-19. The study period, from March to August 2021, coincides with the second wave of the COVID-19 pandemic in India [32, 33]. We found that with an increase in a number of severe COVID-19 cases per household, the risk of facing CHE becomes higher. Having one severe COVID-19 patient in a household increases the risk of facing CHE by fourfold, and having more than one severe COVID-19 patient in a household increases the risk by five times approximately.

A recent study from South India found that 49.7% of households faced CHE, and 32.9% of the households incurred distress financing due to COVID-19 [34]. As documented in east-central India, those hospitalised in public hospitals [3.2% (1.8–5.7%)] incurred CHE more than those hospitalized in private hospitals [58.9% (50.5–66.74%)]. Overall, 20.3% (16.9–24.1%) of those hospitalised had CHE [25], which is more comparable with our study findings. Comorbidities and being elderly were associated with the severity of COVID-19 and the need for hospitalisation [35–38].

According to the World Bank, the out-of-pocket expenditure (OOPE)% of current health expenditure in India was 54.78% for the year 2019 [35]. The present study resulted with an OOPE% of 61.7%, which is closer to the value of the national OOPE% [39]. The mean OOPE for COVID-19 per household in this study, at US $1,643, is different from the findings from other parts of India. A study from Kerala reported the mean OOPE as US $502 during the second wave [34], and in a survey from Chhattisgarh, the mean OOPE was US $2,229 for private and US $64 for government hospitals in the first wave of the COVID-19 pandemic [25]. Our analysis found that households that received insurance co-payments/reimbursement were six times more likely to experience CHE, pointing towards likely ineffectiveness of insurance co-payment/reimbursement, whether public or private. Similar findings from India [25] and other countries such as Peru and USA have reported high OOPE for COVID-19 hospitalization and the ineffectiveness of health insurance [16, 40, 41].

Tamil Nadu State reports that approximately 66.5% of households are covered by the Chief Minister's Comprehensive Health Insurance Scheme (CMCHIS), implemented to provide financial protection to the vulnerable, compared to a national average of 41% of households [42, 43]. However, the NSSO 75th round showed that about 14% of the rural and 19% of the urban population have some health expenditure coverage [17]. Among them, about 13% of the rural and 9% of the urban population were covered by AB-PMJAY [18]. Our findings showed similar observations that one in eight households reported having health insurance. Given that more than a third (66.5%) of the households had only one earning member, and that the median (IQR) household annual income of Rs 300,000 (276,000) is higher than the CMCHIS income cut of Rs. 1,20,000 per person per year [44]. The reported insurance coverage in our study need to be cautiously interpreted considering information bias, recall bias, and whether study participants were aware of being covered under government insurance [45, 46].

The concentration curve of OOPE for increased COVID-19 severe cases in a household shifted upward, suggesting that the concentration of OOPE increased among households having more than one severe case of COVID-19. When using the annual income or subsistence income, it almost coincides with the diagonal line, suggesting that COVID-19 severity is distributed equally among all income groups. The findings suggest that OOPE was concentrated not among poorer households but among households with a more number of severe COVID-19 cases.

Overburdened public hospitals divert individuals to seek treatment from the private sector, and using private hospitals increases the likelihood of catastrophic expenditure [12]. The relative size of out-of-pocket spending has a huge impact on financial risk protection and access to care [26]. India continues to leverage patients' out-of-pocket payments to providers instead of allocating larger budget shares for health care with an increase to finance the health system; it will prevent some people from seeking care and cause financial catastrophe and impoverishment in households that do obtain care. Hence, it is also trivial to judicially use the available public resources to improve access and protect households from financial risk.

Though we attempted to estimate the OOPE and CHE for COVID-19, the study has certain limitations. First, we have not assessed the extent of borrowing and selling of assets due to data limitations. The extent of indebtedness and source of borrowing would have affected the household's welfare in the long run. Second, the direct or indirect expenses and the OOPE reported by the household may have had recall bias. Third, the digital inequities across income levels and the gender gap in phone ownership or the inability to establish a face-to-face rapport impede telephone-based data collection. In addition, participants may have been exposed to interview fatigue as well; however, the telephonic interviews also included pauses and several sessions to reduce interview fatigue. Furthermore, participant over- or under-reporting of subsistence expenditure or income status may have resulted in information bias, which may have resulted in an over- or underestimation of the proportion of CHE and strength of association. Despite these limitations, this study provides an estimate of OOPE and the proportion of households that experienced CHE due to COVID-19 during the second wave in India, which had serious consequences in the form of spiralling cases, reduced supplies of essential treatments, and increased fatalities in the younger age group. Further, this study explores the main drivers of CHE and looks for the association of household characteristics and COVID-19 characteristics with CHE.

## Conclusion

An increase in severe COVID-19 patients in households will upsurge OOPE and the likelihood of households experiencing CHE. The surge of COVID-19 cases during the second wave affected households without insurance, with an increase in the number of hospitalizations for severe COVID-19. Strengthening the health insurance is the need of the hour, as it was not commensurable in financial risk protection of households with severe COVID-19 from CHE. We recommend strengthening public health care and insurance indemnity by increasing government spending to prevent OOPE and CHE. We also recommend that studies be conducted to explore public-funded health insurance awareness, its uptake, utilization, and determinants among Tamil Nadu’s population against the pandemic risk.

## Supplementary Information

**Additional file 1: Supplementary Figure 1.** Concentration curves and indices. **Supplementary Table 1.** Concentration curves and indices. **Supplementary Table 2.** Household Income, Expenditure and Out of pocket Expenditure details. **Supplementary Table 3.** STROBE Checklist.

## Data Availability

The datasets used and analysed during the present study are available from the corresponding author upon reasonable request.
